# Temporal resolution and pitch discrimination in music education: novel data in children

**DOI:** 10.1007/s00405-024-08571-7

**Published:** 2024-04-04

**Authors:** Georgios Psarris, Nikos Eleftheriadis, Christos Sidiras, Afroditi Sereti, Vasiliki Maria Iliadou

**Affiliations:** 1 School of Medicine, Aristotle University of Thessaloniki, 54124 Thessaloníki, Greece; 2 ENT, Private Practice, 54623 Thessaloníki, Greece

**Keywords:** Music education, Auditory processing, Temporal resolution, Temporal processing, Pitch discrimination

## Abstract

**Background:**

Rehabilitation of hearing and listening difficulties through neuroplasticity of the auditory nervous system is a promising technique. Evidence of enhanced auditory processing in adult musicians is often not based on clinical auditory processing tests and is lacking in children with musical education.

**Purpose:**

The aim of this study is to investigate the temporal resolution and frequency discrimination elements of auditory processing both in adults and children with musical education and to compare them with those without any musical education.

**Methods:**

Participants consisted of ten children without musical training and ten children with musical training with mean age 11.3 years and range 8–15 years as well as ten adults without musical education and ten adults with musical education with mean age 38.1 years and range 30–45 years. All participants were tested with two temporal resolution tests (GIN:Gaps-In-Noise and RGDT:Random Gap Detection Test), a temporal ordering frequency test (FPT:Frequency Pattern Test), and a frequency discrimination test (DLF: Different Limen for Frequency).

**Results:**

All test results revealed better performance in both children and adults with musical training for both ears.

**Conclusion:**

A positive effect of formal music education for specific auditory processing elements in both children and adults is documented. Larger samples, longitudinal studies, as well as groups with impaired hearing and/or auditory processing are needed to further substantiate the effect shown.

## Introduction

The ability of the brain to modify, adapt, and form new synapses as a result of life experiences is referred to as brain plasticity or neuroplasticity and is an active process [1]. Music training, as a way to study neuroplasticity, is receiving wide interest from the scientific community [2]. Differences in brain structure and function between musicians and individuals without music training are reported by many correlational studies [3–7]. The differences provide the background for a quicker processing of auditory inputs. In addition, improved performance of musicians in abilities, such as executive function, speech in noise perception, and pitch discrimination, is reported [8–10]. A recent systematic review and meta-analysis [11] of 62 longitudinal studies of healthy individuals shows auditory processing enhancement as a result of music training. The type of music training was diverse (from music education of different musical instruments to chorus, orchestra, ensemble performances, and computer-based music training) with the studies including adults as well as children while having a control non-training group. Pitch discrimination followed by rhythm discrimination were the auditory processing elements that showed the most improvement with high effect sizes.

Speech in noise perception is a core element of auditory processing and is recently recognized as a tool for assessing auditory function beyond the pure-tone audiogram [12]. Auditory neuroplasticity requires intact auditory capacity and improves its more central elements, such as speech in noise perception, pitch pattern recognition, temporal resolution, and dichotic listening [13]. It may be utilized as a foundation for hearing development programs for elderly rehabilitation [14]. EEG studies show that music training may affect numerous areas of auditory processing, including those related to instrumental and pure-tone perception, as well as melody and rhythm perception [15, 16]. Additional research demonstrates that music benefits the transfer of cognitive domains in the pediatric [4, 15] population as musical training enhances sensitivity to tuning, a particular fundamental acoustic parameter that is crucial for both speech and music prosody. This improves children's capacity to identify tuning changes in both language and music [4]. EEG research in children demonstrates neuroplasticity effects [17–20] following even a brief music training period of 4 weeks [17, 18].

Benefits in auditory abilities are consistent with the notion that the auditory system is altered by music training [21] reporting correlational evidence of advantages in abilities, such as rhythm, pitch, and timbre discrimination in musicians [22–24]. Research by Herholz and Zatorre (2012) indicates that active exposure to music can integrate several abilities, contingent on the length of music training, including auditory perception, kinesthetic control, visual perception, and memory encoding, [21]. To investigate various temporal processing capacities, Rammsayer and Altenmuller (2006) discovered that musicians outperformed non-musicians in terms of temporal discrimination, rhythm perception, auditory fusion skills, and tone length perception [22]. Mishra et al., (2014) compared classical musicians to non-musicians, demonstrating that musicians have enhanced sensitivity to detect the presence of a silent temporal gap between two spectrally dissimilar markers [25].

In brief, auditory processing enhancement following music education and training is found to be present in both correlational and longitudinal studies in children and adults. It should be noted that the literature includes either correlation comparative studies between musicians and non-musicians [26] or differences observed as a result of music education and training of a given duration in time [11]. In both cases, few of those studies regarding adults applied the clinically used tests (GIN = Gaps-In-Noise, RGDT = Random Gap Detection Test, and FPT = Frequency Pattern Test) to evaluate temporal resolution and temporal ordering, respectively [26–29]. Comparative evaluation of children with and without musical education using the clinically available tests mentioned above is lacking in published research to the best of our knowledge. Aim of the present study is to compare a group of children with music education with a control non-training group as well as a group of adults with music education with a control group. Comparison of auditory processing will be investigated by the use of clinically available auditory processing tests (GIN, RGDT, and FPT) and one regarding frequency discrimination ability (DLF = Different Limen of Frequency). This later is added to have a more detailed report on frequency discrimination that is known to be highly influenced by exposure to music education.

## Materials and methods

### Participants

A total of 40 participants were evaluated. Twenty of them were children and twenty were adults. Ten children (mean age 11.3 years, range 8–15 years) with music education were recruited from a local music conservatory based on more than 2 years of formal music education with a frequency of two times a week and upon their availability to serve as experimental group. Ten children (mean age 11.3 years, range 8–15 years) with no formal music education were recruited from local schools to serve as controls. It should be noted that all children attending elementary and high school attend music lessons within the school framework between 1 and 2 h per week. Ten music-educated adults serving as the experimental adult group (mean age 38.1 years, range 30–45 years) were recruited from a local conservatory with the inclusion criterion of taking lessons for more than 5 years. Ten adults without music training (mean age 38.1 years, range 30–45 years) were recruited from the family and friendship context to serve as controls. These adults had not received any formal music training apart from that which they received as students in the context of the school. We are defining formal music education as one acquired in conservatories and the participants in both the adult and the children’s group have been in different classes while learning different instruments.

All participants were native Greek speakers. Subject inclusion criteria were as follows: (1) all individuals had to have hearing thresholds less than 20 dB HL at all frequencies tested for children participants and less than 25 dB HL for adults’ participants (octave frequencies between 0.25 and 8 kHz); (2) no documented audiological or neurological disorder. Children in the experimental groups had at least 2 years of music education and adults had more than 5 years of music education (Table 1). Control groups for both children and adults had no music education. All individuals in the study provided informed consent and parents provided additional informed consent for their children. The study was approved by the bioethics committee of the authors' affiliated Medical School (6.607; 14.06.2022).

**Table 1:** Samples’ size and formal music education (in years) of participants

	*N*	Formal music education (in years)
Control	10	0 years
Children		
Experimental	10	> 2 years
Control	10	0 years
Adults		
Experimental	10	> 5 years
TOTAL	40	

Τhe assessment of the participants took place in a quiet speech therapy conference room. Τhe assessment of the children was done in two different sessions of approximately one hour each, while the assessment of the adult participants in one session of approximately 2 h. Τhe order in which the tests were administered was of decreasing difficulty. At the beginning, the two tests of temporal analysis were administered (GIN and RGDT), while the next two tests concerned pitch and frequency discrimination (FPT and DLF). Between tests there was a break of about 10 min where the participants remained in the evaluation area. The groups of children and especially adults with music training were the ones that needed the shortest breaks. Verbal reward for following instructions was given at regular intervals to maintain higher levels of cooperation and attention. In addition, promised access to its individual's performance upon completion of the evaluation created a strong motivation.

### Testing

*Frequency Pattern Test* Frequency Pattern Test is a psychoacoustic test consisting of a series of three tone burst patterns at two different frequencies, i.e., 1122 Hz high frequency) and 880 Hz (low frequency). The duration of each tone is 200 ms with an inter-stimulus interval of 150 ms and a rise–fall time of 10 ms [30, 31]. We administered the test at 50 dB HL monaurally—through headphones (Sennheiser HD-280 Pro). The test has 60 test items for each ear. The first six items are used for practice. The individuals tested were asked to label each item of the pattern using the words Low (L) and High (H) and repeat the order they were hearing. There were six possible combinations of tones (LLH, LHL, LHH, HLH, HLL, and HHL). Based on the participants' responses, a percentage of the correctly identifies patterns per ear is documented. For the Frequency Pattern Test, 75% and above are considered to be typical test results for individuals aged 11 and over.

*Gaps-In-Noise Test* Gaps in Noise is a clinical measure of temporal resolution administered monaurally [32]. Its practice section consists of 10 trials of randomly presented gaps with varying duration embedded in white noise. The main test has four lists with gap durations ranging from 2 to 20 ms; embedded in 6 s of white noise. Some trials have no gaps. Location of the gaps is varied. The duration of the gaps is 2, 3, 4, 5, 6, 8, 10, 12, 15, and 20 ms. Each gap occurs six times within each list. The purpose of this test is to determine the gap detection threshold in msec. The gap detection threshold is the smallest interval detected in at least 4 out of 6 presentations of a given duration [32, 33].

We administered the test at 50 dB HL monaurally—separately for each ear [34]. GIN consists of a training-familiarity list and 4 test lists of 32 to 36 trials each. We instructed participants to raise their hand each time a gap was detected. For analysis, the approximative threshold (ATh) was employed. The ATh was found to be the shortest gap duration for which at least four out of six identifications were accurate (67%) [32]. For gaps that lasted longer, this performance level had to be preserved or increased.

*Random Gap Detection Test* Random Gap Detection Test (RGDT) was the second test of temporal resolution employed [35]. A number of pairs of pure tones at frequencies of 500 Hz, 1000 Hz, 2000 Hz, and 4000 Hz are presented using the RGDT technique, with the time intervals between each stimulus varied at random between 0 and 40 ms (0, 2, 5, 10, 15, 20, 25, 30, and 40). Inter-trial time is set at 4.5 s to provide subjects enough time to reply. The stimulus had a duration of 17 ms, a rise and fall time of 1 ms. There is a practice tract that has a gradual increase of time interval between the two pure tones, starting with zero and going up to 40 ms. Τhe test was presented at 50 dB HL at a binaural condition via headphones.

Individuals were asked to say whether they heard one or two stimuli for each trial. The practice tract is not scored. The overall gap detection threshold is computed for a total of 36 trials [35]. For each frequency tested, the threshold of gap detection is determined as the shortest time interval at which the subject reports hearing two tones. The overall score is the mean of the four tested frequencies.

*Different Limen of Frequency* Discrimination tasks are an important component of central auditory testing. The available clinical tests focus more on pattern recognition of specific frequencies than on frequency threshold. Due to this, we created an adaptive behavioral DLF test with tone stimuli digitally generated using Audacity 3.2.1.

*Procedure* Τhe test was presented at 50 dB HL in binaural condition via headphones. Two stimuli were presented in each trial; the base frequency being 1 kHz tone. Based on previous study, each tone frequency had a length of 350 ms [36]. The gap between the baseline frequency and the test frequency was set at 300 ms. Six changes were introduced into the test stimuli as a percentage geometric progression of the fundamental frequency and one no-change stimulus was added: 1.56%, 3.12%, 6.24%, 12.48%, 25%, and 50%. Stimulus conditions and the step size are listed in Table 2.

**Table 2:** Stimulus conditions and step size of the fundamental frequency

Percent change in %	Frequency in Hz	Frequency in Hz
0	1000	1000
1.56	1000	1015.6
3.12	1000	1031.2
6.24	1000	1062.4
12.48	1000	1124.8
25	1000	1250
50	1000	1500

Both descending and ascending techniques were used. We started using a descending run, with the first pair of tones 1500 Hz and 1000 Hz. The individual's response was to answer whether the tones are the “same” or “different”. When tones were recognized as different in two consecutive trials, the stimulus level for the following trial was lowered as a percentage geometric progression of the fundamental frequency (the step size). This process was carried out repeatedly until the individual characterized the two tones as "the same" (point 1). We continued with an ascending run. In the ascending run, presentation was stopped when individuals characterized the two tones as "different" (point 2). Since cross over between hearing and not hearing lies somewhere between the lowest audible level and the highest inaudible level, threshold expressed in Hz for each series was taken as the average point between crossover of descending and ascending run (point 1, point 2).

### Statistical analysis

Data were analyzed using SPSS 28. As a first step, distribution of data was evaluated. In terms of the skewness and kurtosis requirement, all data were normally distributed, with z values ranging from – 1.96 to + 1.96 [37]. Parametric statistics were applied (Student’s *T* test–Independent samples *t* test). The level of significance for statistical tests was *p* < 0.05. Correlation analysis of variables was done using the Pearson correlation efficient.

## Results

**Fig. 1:**
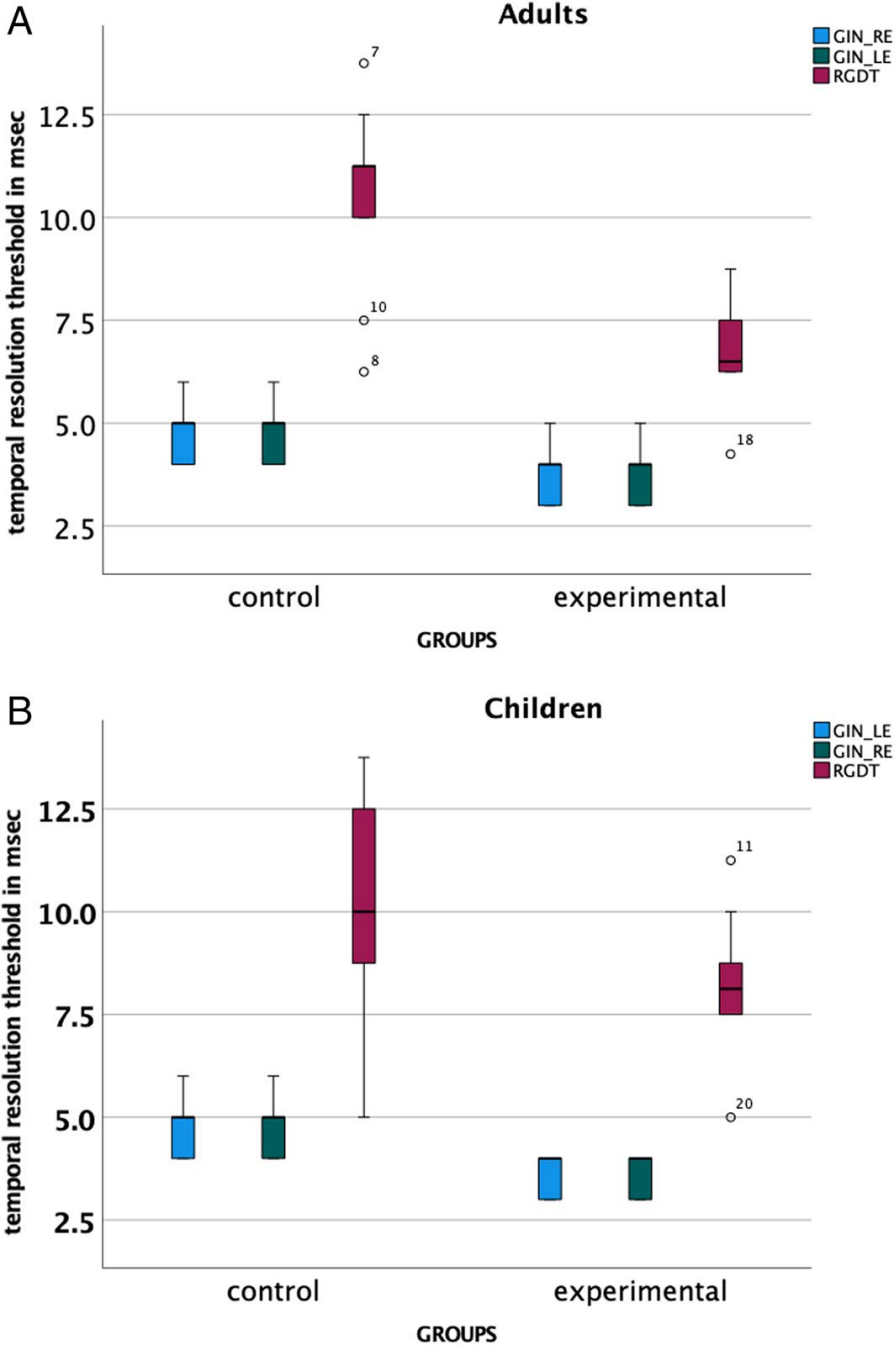
XXX

Student’s *T *test was run for GIN_RE (Right Ear), GIN_LE (Left Ear), RGDT, FPT_RE, FPT_LE, and DLF comparing the means across the groups of children and adults for temporal resolution, temporal processing, and frequency discrimination. The analysis revealed a statistically significant better performance in the experimental group for the variables of GIN_RE, GIN_LE, FPT_RE, FTP_LE, and DLF in comparison to the control group in both children and adults. A statistically significant lower threshold was documented for the RGDT of the experimental as compared to the control group in adults but not in children (Fig. 1A, B). In both children and adults (with the exception of RGDT in children) performances across temporal resolution, temporal processing and frequency discrimination were better for those with a formal music education as opposed to normal controls.

### Temporal resolution (GIN and RGDT)

GIN Results Student’s *T* test was run for GIN_RE (GIN Right Ears) and GIN_LE (GIN Left Ears). The analysis revealed a statistically significant better performance (Table 3) in terms of lower gap detection threshold of the experimental compared to the control group in both children (right ears *t* = 3.76, *p* < 0.001, left ears *t* = 3.76, *p* < 0.001) and adults (right ears *t* = 4.9, *p* < 0.001, left ears *t* = 4.26, *p* < 0.001).

**Table 3:** Means, standard deviations, and *p* values of the two temporal resolution tests in msec (RGDT & GIN), the frequency pattern test (FPT) in percent correct and the frequency discrimination test in Hz (DLF) in both control and experimental groups in children and adults are presented

	GIN_RE	GIN_LE	RGDT	FPT_RE	FPT_LE	DLF
Mean (SD)						
Children						
Control	4.8 (0.79)	4.8 (0.79)	10.25 (2.48)	76.59 (4.15)	76.25 (4.28)	55.38 (25.32)
Experimental	3.7 (0.48)	3.7 (0.48)	8.25 (1.68)	84.24 (5.67)	85.91 (4.38)	17.16 (8.05)
Student’s *t *test *p*	0.001	0.001	0.05	< 0.05	< 0.001	< 0.001
Mean (SD)						
Adults						
Control	5.9 (1.28)	5.9 (1.28)	10.5 (2.22)	79.92 (5.2)	78.92 (6.66)	10.92 (6.57)
Experimental	3.7 (0.67)	3.9 (0.74)	6.72 (1.19)	93.24 (2.22)	93.07 (2.65)	5.46 (3.76)
Student’s *t* test *p*	< 0.001	< 0.001	< 0.001	< 0.001	< 0.001	< 0.05

The values inside the parentheses are the standard deviations of the data distribution

*RE* right ear, *LE* left ear

RGDT Results Student’s *T *test was run for RGDT thresholds. The analysis revealed a statistically significant difference between the experimental and the control group in adults (*t* = 4.73, *p* < 0.001). Specifically, adults with music education had a lower mean score in the RGDT test (i.e., better gap detection threshold) than adults without any music education (Table 3, Fig. 1A). No statistically significant difference was found for children (*t* = 2.1, *p* = 0.05).

### Pitch discrimination (FPT and DLF)

#### FPT Results 

Student’s *T* test was run for FPT_RE (FPT Right Ears) and FPT_LE (FPT Left Ears). The analysis revealed statistically significant higher mean scores (Table 3) for the experimental compared to the control group in children (right ears *t* = -3.44, *p* = 0.003, left ears *t* = – 4.98, *p* < 0.001). Additionally, the analysis revealed statistically significant higher mean scores (Table 3) for the experimental compared to the control group in adults (right ears *t* = – 7.44, p < 0.001, left ears *t* = – 6.23, *p* < 0.001).

#### DLF Results

Student’s *T* test was run for DLF. The analysis revealed a statistically significant better discrimination performance (Table 3) for the experiment compared to the control group in both children and adults, respectively (*t* = 4.54, *p* < 0.001; *t* = 2.8, *p* = 0,035). DLF scores are expressed in Hertz, and lower DLF values indicate better discrimination performance. Children in the experimental group could discriminate between the two tones at a mean of 17.16 Ηz (SD = 8.05), whereas children in the control group could discriminate between the two tones at a mean of 55.38 Hz (SD = 25.32). Adults in the experimental group could discriminate between the two tones at a mean of 5.46 Ηz (SD = 3.76) whereas adults in the control group could discriminate between the two tones at a mean of 10.92 Hz (SD = 6.57).

## Correlations between FPT AND DLF values

Pearson test was run for DLF and FPT_RE (FPT Right Ear) and FPT_LE (FPT Left Ear) in children and adults. The analysis revealed a statistically significant negative correlation for children (*r* =  − 0.621, *p* < 0.01; *r* =  − 0.717, *p* < 0.01 between DLF and FPT_RE; DLF and FPT_LE, respectively; see Table 4). Analysis of adults' results showed a statistically significant negative correlation (*r* =  − 0.528, *p* < 0.01; *r* =  − 0.571, *p* < 0.01 between DLF and FPT_RE; DLF and FPT_LE, respectively; Table 4). A total correlation analysis of all participants, adults and children, revealed an even stronger correlation (*r* =  − 0.584, *p* < 0.001; *r* =  − 0.579, *p* < 0.001 between DLF and FPT_RE; DLF and FPT_LE, respectively; Table 4). Lower thresholds on DLF tend to coincide with high scores on FPT, and vice versa.

**Table 4:** Correlation analysis of children; adults; children and adults (DLF: Different Limen of Frequency, FPT_RE: Frequency Pattern Test Right Ear, FPT_LE: Frequency Pattern Test Left Ear)

Variables	DLF	FPT_RE	FPR_LE
Correlation analysis of children			
1. DLF		– 0.621**	– 0.717**
2. FPT_RE	– 0.621**		0.920**
3. FPT_LE	– 0.717**	0.920**	
**p < 0.01; *N *= 20			
Correlation analysis of adults			
1. DLF		– 0.528**	– 0.571**
2. FPT_RE	– 0.528**		0.946**
3. FPT_LE	– 0.571**	0.946**	
**p < 0.01; *N* = 20			
Correlation analysis of children and adults			
1. DLF		– 0.584***	– 0.579***
2. FPT_RE	– 0.584***		0.939***
3. FPT_LE	– 0.579***	0.939***	
*p* < 0.001; *N* = 40			

## Discussion

Aim of the present study was to compare a group of children with music education with a control non-training group as well as a group of adults with music education with a control group. Both experimental groups performed better in temporal resolution when compared to the respective control group with the exception of children's RGDT. The later had a p value equal to 0.05, very close to reaching statistical significance between groups. This documentation of higher temporal resolution in individuals with music education is consistent with earlier research indicating that musicians have better performance [14, 25]. In our study, the mean gap detection threshold was 3.7 ms for the right ears and 3.9 ms for the left ears for the experimental group of adults with a 2.2 ms and 2 ms difference, respectively, compared to the control group. Our findings agree with other researchers’ findings [38] that musicians have an average of 2 ms shorter gap detection thresholds on the GIN test (raw values 3.5 ms both for right and left ears) than their non-musician counterparts. Research showing the same effect in children is scarce. Sangamanatha et al. (2012) reported that children with music training performed at par with adults without music training, and better than children with no music training on temporal resolution [39].

Our novel findings for gap detection thresholds evaluated by the GIN test documented that children with music education performed better than adults without music education (3.7 ms vs 5.9 ms). They had more efficient temporal resolution than the children in the control group. The raw score of the temporal resolution measured by the clinical tool of GIN is very close to the 3.13 ms threshold documented by Sangamanatha et al. [39] using an experimental procedure for gaps in noise identification in children. This indicates consistency beyond the type of test used (clinical vs experimental). To the best of our knowledge, there is a lack of literature for mean values in children with music education evaluated by RDGT. Our findings for adults agree with the results by Kahraman et al. (2021) [27] and demonstrate that musicians had a statistically significant lower threshold of gap identification than non-musicians.

Music training appears to improve pitch perception [40, 41]. Frequency discrimination evaluated with two tests in our study (FPT & DLF) was found to be statistically significant better in both children and adults. FPT reports on temporal ordering as well. To the best of our knowledge, these results of the present study for children are novel. The findings of our research for adults are confirmed by previous studies [27, 42]. Nascimento et al. (2010) administered the FPT on 20 violinists and 20 non-musicians and found statistically better performance on FPT in musicians [45]. In a recent study by Kahraman et al. (2021) musicians between 20 and 40 years had 88.83% correct answers for right ear and 89.33% for left ear on FPT and they performed statistically better in comparison to no musicians group [27]. Their findings are slightly lower than our results for adults with music education. Majak et al. (2016) compare the performance of FPT in normal hearing musicians and non-musicians. In contrast to our findings, they found that musicians had an excellent performance on FPT with 100% correct answers in both ears [28]. The mean age of the musicians group in their study was 21.4 years, in contrast to our study that was 38.1 years and that is a parameter that may explain the excellent values of the youngest participants. Karimi et al. (2018) compared the performance of adults with 8 years of music education vs adults without music education (ages between 21 and 44 years) on FPT. The results revealed that the FPT score in both ears differed significantly between the two groups. Musicians performed significantly better than non-musicians [43].

DLF was used in our study along with FPT as a more true evaluation of frequency discrimination compared to the FPT having added aspects of temporal processing in terms of pattern recognition. Both tests were able to document better performance with music education across the life span. Our findings are in accordance with the results of Kishon-Rabin et al. (2001) [44] who found that mean frequency discrimination thresholds for adult musicians were approximately half the values of the non-musicians and by other studies that shows musicians are able to discriminate at much lower thresholds than non-musicians [10, 45, 46]. Kishon-Rabin et al. (2001) [44] state immediate frequency discrimination threshold improvement for both musicians and non-musicians following relatively brief training, indicating that the frequency discrimination task is trainable. Additionally, vocal musicians performed significantly better compared with non-musicians on frequency discrimination thresholds [47]. Eight-year-old children with music education have higher auditory temporal-interval discrimination as well as frequency discrimination, which is correlated with reading skills [48].

Correlation between DLF (frequency discrimination threshold) and FPT (frequency discrimination & temporal processing) was statistically significant and revealed that FPT has a strong frequency discrimination component. DLF and FPT were correlated for children and adults (*p* < 0.01), with correlation being stronger (*p* < 0.001) when the analysis was done for all 40 individuals across the life span. This might indicate that both tests have much more in common, even though they clearly have methodological differences in terms of being adaptive or not, being forced or not and having different stimuli configurations. Our findings are not in full agreement with Flagge et al. (2020) who found a moderate correlation between the DLF and FPT values for children 6;11–11;3 years old [49]. The stronger correlation of the present study might be the result of the different standard frequency used by Flagge et al., 220 Hz as opposed to the use of 1 kHz in our study.

This study’s results show a positive effect of formal music education for specific auditory processing elements in both children and adults. Larger samples as well as longitudinal studies are needed to further substantiate the effect shown.

## Conclusion

In conclusion, results of this study suggest that music education provides a significant benefit in auditory processing across the life span. The benefits documented are in temporal resolution and pitch discrimination. Larger samples of children with music education as well as groups with impaired hearing and/or auditory processing are needed to verify that music education may be used as a rehabilitation tool for hearing difficulties to improve auditory processing.

## Data Availability

Data are available on special request when contacting the corresponding author.

## Declarations

### Availability of supporting data

Upon reasonable request, the data will be made available.
